# Supplementary material for: Violent deaths following disasters: A retrospective analysis
Source: PLoS One. 2025 Dec 4;20(12):e0337968. doi: 10.1371/journal.pone.0337968 (PMC12677568; doi:10.1371/journal.pone.0337968)
Supplement: S1 Table — (DOCX) [file pone.0337968.s001.docx]

|  | All Violent Deaths | | | | | | | |
| --- | --- | --- | --- | --- | --- | --- | --- | --- |
|  | **Pre-Disaster** | | | | **Disaster** | | | |
| State | **No PA** | | **Public Assistance** | | **No PA** | | **Public Assistance** | |
|  | **IR** | **95% CI** | **IR** | **95% CI** | **IR** | **95% CI** | **IR** | **95% CI** |
| CO | 3.945 | 3.369 – 4.522 | 6.129 | 4.297 – 7.961 | 4.800 | 4.164 – 5.436 | 5.702 | 3.935 – 7.469 |
| NC | 4.719 | 4.244 – 5.195 | 5.462 | 3.726 – 7.199 | 4.332 | 3.877 – 4.788 | 3.594 | 2.185 – 5.002 |
| OK | 4.193 | 3.545 – 4.841 | 6.604 | 1.320 – 11.888 | 4.532 | 3.858 – 5.205 | 3.302 | 0.000 – 7.085 |
| OR | 4.389 | 3.614 – 5.165 | 2.826 | 1.740 – 3.912 | 3.354 | 2.676 – 4.033 | 3.043 | 1.916 – 4.170 |
| WI | 3.421 | 2.931 – 3.911 | 3.532 | 1.225 – 5.840 | 4.153 | 3.613 – 4.693 | 5.495 | 2.616 – 8.373 |
| All | 4.169 | 3.914 – 4.424 | 4.581 | 3.768 – 5.394 | 4.299 | 4.040 – 4.558 | 4.131 | 3.356 – 4.903 |

**S1 Table.** Rates for all violent deaths in pre-disaster and disaster periods stratified public assistance eligibility.
