# Supplementary material for: Violent deaths following disasters: A retrospective analysis
Source: PLoS One. 2025 Dec 4;20(12):e0337968. doi: 10.1371/journal.pone.0337968 (PMC12677568; doi:10.1371/journal.pone.0337968)
Supplement: S2 Table — (DOCX) [file pone.0337968.s002.docx]

|  | Suicides | | | | | | | |
| --- | --- | --- | --- | --- | --- | --- | --- | --- |
|  | **Pre-Disaster** | | | | **Disaster** | | | |
| State | **No PA** | | **Public Assistance** | | **No PA** | | **Public Assistance** | |
|  | **IR** | **95% CI** | **IR** | **95% CI** | **IR** | **95% CI** | **IR** | **95% CI** |
| CO | 3.375 | 2.842 – 3.908 | 5.274 | 3.575 – 6.974 | 4.099 | 3.511 – 4.686 | 4.134 | 2.629 – 5.638 |
| NC | 2.672 | 2.314 – 3.030 | 4.456 | 2.888 – 6.025 | 2.385 | 2.046 – 2.723 | 2.731 | 1.503 – 3.959 |
| OK | 2.865 | 2.330 – 3.400 | 6.604 | 1.320 – 11.888 | 3.438 | 2.851 – 4.024 | 3.302 | 0.000 – 7.085 |
| OR | 3.640 | 2.934 – 4.346 | 2.608 | 1.565 – 3.652 | 2.748 | 2.134 – 3.362 | 2.934 | 1.828 – 4.041 |
| WI | 2.945 | 2.490 – 3.400 | 3.532 | 1.225 – 5.840 | 2.982 | 2.524 – 3.440 | 3.532 | 1.225 – 5.840 |
| All | 3.002 | 2.786 – 3.219 | 4.018 | 3.257 – 4.779 | 3.039 | 2.821 – 3.256 | 3.267 | 2.581 – 3.954 |

**S2 Table.** Rates for suicide deaths in pre-disaster and disaster periods stratified public assistance eligibility.
