# Supplementary material for: Violent deaths following disasters: A retrospective analysis
Source: PLoS One. 2025 Dec 4;20(12):e0337968. doi: 10.1371/journal.pone.0337968 (PMC12677568; doi:10.1371/journal.pone.0337968)
Supplement: S3 Table — (DOCX) [file pone.0337968.s003.docx]

|  | Other | | | | | | | |
| --- | --- | --- | --- | --- | --- | --- | --- | --- |
|  | **Pre-Disaster** | | | | **Disaster** | | | |
| State | **No PA** | | **Public Assistance** | | **No PA** | | **Public Assistance** | |
|  | **IR** | **95% CI** | **IR** | **95% CI** | **IR** | **95% CI** | **IR** | **95% CI** |
| CO | 0.570 | 0.351 – 0.789 | 0.855 | 0.171 – 1.540 | 0.701 | 0.458 – 0.944 | 1.568 | 0.641 – 2.495 |
| NC | 2.048 | 1.734 – 2.361 | 1.001 | 0.261 – 1.752 | 1.948 | 1.642 – 2.253 | 0.863 | 0.172 – 1.553 |
| OK | 1.328 | 0.964 – 1.693 | xx | xx - xx | 1.094 | 0.763 – 1.425 | xx | xx - xx |
| OR | 0.749 | 0.429 – 1.070 | 0.217 | 0.000 – 0.517 | 0.607 | 0.318 – 0.895 | 0.109 | 0.000 – 0.322 |
| WI | 0.476 | 0.293 – 0.659 | xx | xx - xx | 1.171 | 0.884 – 1.458 | 1.962 | 0.242 – 3.683 |
| All | 1.167 | 1.032 – 1.302 | 0.563 | 0.278 – 0.848 | 1.260 | 1.120 – 1.400 | 0.864 | 0.512 – 1.217 |

**S3 Table.** Rates for other violent deaths in pre-disaster and disaster periods stratified public assistance eligibility.
