# Supplementary material for: Violent deaths following disasters: A retrospective analysis
Source: PLoS One. 2025 Dec 4;20(12):e0337968. doi: 10.1371/journal.pone.0337968 (PMC12677568; doi:10.1371/journal.pone.0337968)
Supplement: Attachment — Submitted filename: Response to Reviewers.docx [file pone.0337968.s005.docx]

We thank the reviewers for the substantive responses. We have addressed these comments below in blue text.

**Reviewer #1:** Thank you for the opportunity to review this paper. This is a neat and to-the-point study showing inconclusive results, but what makes the paper interesting is that the authors tried to address an important research gap in a creative way using existing data. What I feel is that the authors should explore this more in depth and reflect more on the methodological choices and discuss them more thoroughly, as this can pave the way for future studies that may yield more conclusive results. I have noted some comments aligned with this:

**Methodology:**

- The study design is not clearly presented. Perhaps adding a study schematic showing how the temporal association was assessed would be helpful.
  - Figure 1 has been added to visually show the temporality of 3-months before and 3-months after the initial disaster event date, and additional clarification has been added in-text, reading: *“The disaster period covered the three months – operationalized as calendar months – following the disaster start date, as defined in the federal disaster declaration.”*
- Suggest to clearly state (with e.g. sub-sections) what is the main outcome of interest, and eventually secondary outcomes of interest.
  - Subsections and explicit definitions have been added under the *Analysis* section of Methods and Materials. The methods section and results have been updated following updated analysis in response to reviewers.
- What are problems or advantages of putting all violent deaths together? (to consider for limitations).
  - We have addressed this in two ways: 1) results for all deaths, as well as stratified by violent death category (i.e., suicide versus other violent death), are presented; and 2) added a brief discussion in the discussion/limitations.
- Clarify the exposure and control. 3 months (what threshold was used operationally , 90 days?), before and after a federally declared disaster.
  - Figure 1 has been added to visually show the temporality of 3-months before and 3-months after the initial disaster event date. These periods were defined as calendar months before and after the date of disaster onset. Federal disaster declarations can happen in real-time, but they are often issued post-event after significant damage is reported or accrued. Therefore, we define the start of the disaster period as the event date included in the declaration rather than the date of the declaration. The text under *Measures* now reads: “*The disaster period covered the three months – operationalized as calendar months – following the disaster start date, as defined in the federal disaster declaration.”*
- Any information on what qualifies as a federal disaster?
  - The following text has been added to define a federally-declared disaster and to clarify the designation of eligibility for public assistance under a federal disaster declaration: *“Under the Stafford Act, state governors and tribal executives can request federal aid to address materiel, recovery, and economic needs, among others, that surpass local, state, or tribal capacity to address these needs (25). Upon an event being declared a major disaster or emergency by the President of the United States, public assistance grants are disseminated through the U.S. Federal Emergency Management Agency (FEMA) to aid in community and public agency response and recovery efforts (26).”*
- Table IV formatting makes it very difficult to read
  - The table orientation has been flipped to improve readability.
- IRRs were calculated, but this implies that incidence rates were calculated, but the methods only talk about a population denominator offset . Does this mean cases per person-time was not calculated? If calculated, please add as appendix.
  - Rates have been submitted as an appendix.

**Discussion:**

- Would have appreciated more in-depth discussion of the methodological choices of this study and put them in context with other existing studies (?) assessing a temporal association between disasters and any potential longer-term effects.
  - We have added more explicit background on the lack of standard timeframe considered in studies of mental health impacts of disasters (see lines 80 – 103 under *Mental health and disasters* in the Background).
- The limitations section is too short and not correctly addressing all potential biases or confounding.
  - Both the discussion and limitations section have been expanded to address this.

**Reviewer #2:** This paper provides an empirical analysis examining the impact of natural disasters on violent deaths across five diverse states, each experiencing distinct natural disaster events. Using county-level data, the paper compares violent death counts—including suicides and non-suicide violent deaths—before and after disaster events in both disaster-affected and unaffected counties. The authors adopt a descriptive analytic approach, contrasting patterns of violent deaths pre- and post-disaster across these groups. However, the robustness and clarity of the findings could be significantly enhanced by explicitly employing a rigorous quasi-experimental design, such as a difference-in-differences (DiD) or event-study framework. Baker et al. (2022) and Obradovich et al. (2018) provide valuable examples of how to implement these methodologies effectively in related contexts.

- We appreciate the reviewer noting the value and added rigor of quasi-experimental approaches for assessing changes in a population outcome from before to after a disaster event. We started with a staggered difference-in-differences approach, but we ultimately had to pivot to a simpler analytical approach to better match the quality, completeness, and sample size of our data.

In addition, the discussion of results (e.g., increased incidence of violent deaths post-disaster in affected versus unaffected areas) would benefit from greater consideration of potential spillover effects between counties, a critical factor given the complexity of post-disaster community dynamics.

- We thank the reviewer for bringing up this important topic (see response below). We have not used the term ‘spillover’ because this term has a very different meaning in public health and epidemiology (e.g., when a novel host population comes in contact with a population with a high prevalence of a given pathogen; often in terms of emerging infectious diseases).

Moreover, several aspects of the methodological and analytical choices require further clarity and justification—for instance, the rationale for selecting a three-month period as representative of short-term mental health impacts, and the specific inclusion and treatment of age as a continuous covariate in regression analyses. The manuscript would benefit from improved precision and clarity in reporting statistical methods and results. Specifically, authors should clarify the statistical tests used, implement appropriate adjustments for multiple testing, and explicitly define their regression modeling approach. These refinements would substantially strengthen the manuscript's rigor and interpretability.

Regarding multiple testing, we respectfully disagree. We are testing one *a priori* stated hypothesis in our analysis – that is comparing the differences in violent death rates for counties that have experienced a disaster from pre- to post-disaster by eligibility for public assistance. The only additional analysis included was stratification by state to account for the effect modification due to characteristics of the state and/or the specific hazard/hazard type. The methods section has been edited to improve clarity. For responses related to the choice of 3-month period, additional details regarding multiple testing adjustments, and treatment of age as a continuous variable, please refer to additional responses below.

**Major comments:**

1. Improve the statistical design of this study by employing a quasi-experimental design, such as difference-in-difference or event-study frameworks.

   Here is a helpful background resource:
   Baker, A. C., Larcker, D. F., & Wang, C. C. (2022). How much should we trust staggered difference-in-differences estimates? Journal of Financial Economics, 144(2), 370-395.

   Here is a study that applies this methodology to look at the impacts of Katrina on mental health in affected and unaffected counties:
   Obradovich, N., Migliorini, R., Paulus, M. P., & Rahwan, I. (2018). Empirical evidence of mental health risks posed by climate change. Proceedings of the National Academy of Sciences, 115(43), 10953-10958.

We thank the reviewer for this suggestion. Our initial model utilized staggered difference-in-difference estimation, but the underlying data were not conducive to model convergence. Therefore, we sought to address our study question using a more straightforward/simple statistical approach which was better suited to the limitations of the underlying data. In future studies, we hope to have more complete and robust data to use the planned staggered DiD estimation. However, the methods section and results have been updated following updated analysis in response to reviewers.

2. Page: 18 lines 220-224: Improve robustness of the discussion of the increased incidence of violent deaths post-disaster in affected and unaffected areas. Please also discuss spillover effects across counties post-disaster.

We have added a discussion of the potential for the spillover of negative social and economic ramifications of disasters for those not eligible for federal public assistance in the third paragraph of the discussion. As noted above, we chose to use an alternative to “spillover” given its different meaning in economics versus epidemiology.

**Minor comments:**

- **Page: 11 lines 76-79**: This passage is difficult to follow. Please expand on the community impacts in the context of disasters. It is also unclear what the Australian experience is contradicting to.
  - This paragraph has been edited for clarity and to provide more background on the temporal considerations for mental health impacts following disasters.
- **Page: 12 line 107:** Expand this discussion to extreme weather exposure and violence literature:

Ranson, M., 2014. Crime, weather, and climate change. J. Environ. Econ. Manag. 67, 274–302. <https://doi.org/10.1016/j.jeem.2013.11.008>

Hsiang, S., Kopp, R., Jina, A., Rising, J., Delgado, M., Mohan, S., Rasmussen, D.J., Muir-Wood, R., Wilson, P., Oppenheimer, M., Larsen, K., and Houser T. (2017). Estimating economic damage from climate change in the United States, Science, 356, 1362–1369

- Additional text has been added to address this concern, reading: “Increasing economic inequality, driven in part by more frequent and severe disasters resulting from climate-related hazards can further exacerbate the negative mental health impacts these events.”
- Additional text has been added to address this concern, reading: “While the relationship between natural hazard exposure and death by homicide or suicide is not well established, it is clear that natural hazard exposure increases perpetration of intimate partner violence and family violence (20) and extreme heat exposure increases a the incidence of various violent behaviors (21).”
- **Page: 14 lines 135-137**: Please support the specific selection of three months to represent short-term mental health effects. Are the results sensitive to the specific choice of the timeframe?
  - Additional references/background has been added to the *Mental health and disasters* section of the Background to better illustrate the wide range of timelines considered throughout the literature.
  - We selected the 3-month time frame in line with the findings of Pietrzak et al., 2012. Added text reads: “Pietrzak et al., (2012), assessed mental health disorders following Hurricane Ike in Galveston and Chambers counties, Texas, at four time points, and found that post-traumatic stress was most prevalent between 2- and 5-months following the event.”
  - We have stated more explicitly that findings are not generalizable to other time frames, and future work should investigate violent deaths at different temporal intervals.
- **Page: 15 lines 151-152**: Earlier you note that age was treated as a continuous variable. How was it introduced in the Poisson regression of aggregate counts in a county/time period?
  - Age was not introduced into the regression model; it is only included in the descriptive table to show the underlying characteristics of all deaths.
  - Age was treated as a continuous rather than categorical variable for descriptive statistics due to the suppression requirements from CDC that would have rendered reported age categories uninformative.
- **Page: 15 lines 154-156:** Provide additional information on the regression analysis performed. What was the unit of observation? What controls, beyond affected status and pre/ post time period were included?
  - Subsections and explicit definitions have been added under the *Analysis* section of Methods and Materials.
- **Page: 16 lines 178-181**: Please elaborate on what the differences were and also in which period (pre/post) these differences were noted.
  - This has been updated to reflect the results.
- **Page: 16 lines 193-194:** Please eliminate discussion of results that did not show up as significant patterns. If a result is not statistically significant, we cannot make claims about protectiveness. Given these wide confidence intervals, it just as easily found be null or >1.
  - This has been updated to reflect the results; additionally, both the methods section and results have been updated following updated analysis in response to reviewers.
- **Page: 26 Table I:** Report the total number of counties in the state.
  - Total number of counties for each state has been added to Table I.
- **Page: 27 Table II:** Add crude incidence rates to this table.
  - This table simply shows counts; incidence rates are shown in Table IV. In response to reviewer 1, we have included crude rates in an appendix.
- **Page: 28 Table III:** Improve labeling. Which test is Chi-Square, and which test is Kruskal-Wallis? Also, apply controls for the multiple testing to the results in this table.
  - The “*Kruskal-Wallis Chi-Square p-value” corresponds with the p-values “0.521*” “0.882*). These are the only Kruskal-Wallis p-values in the table, used to test differences in medians between those with and without public assistance. Additional clarification has been added to Table III.
  - The use of chi-square p-values for categorical variables and the Kruskal-Wallis chi-square p-value for median age is now clarified in the text, reading: “To compare deaths among those eligible for public assistance and those not eligible in pre-disaster and disaster periods, chi-square p-values are reported for categorical variables (i.e., race, sex, marital status, and education), and Kruskal-Wallis chi-square p-values are reported for median age and interquartile range.”
  - Table III only shows descriptive, not inferential, statistics, so there are no controls for multiple testing to be applied. We have changed the title of the table to better reflect the results shown: “Table III. Descriptive statistics of sociodemographic characteristics of all deaths.”
- **Page: 29 Table IV:** Apply adjustments for multiple testing in this table.
  - We thank the reviewer for this comment. We have presented stratified estimates in-line with the stated a-priori research questions: “The aims of this work are 1) to characterize violent deaths in non-disaster and disaster periods and 2) to assess changes in incidence of violent deaths for counties eligible and ineligible for public assistance in non-disaster and disaster periods.”
