# Supplementary material for: Violent deaths following disasters: A retrospective analysis
Source: PLoS One. 2025 Dec 4;20(12):e0337968. doi: 10.1371/journal.pone.0337968 (PMC12677568; doi:10.1371/journal.pone.0337968)
Supplement: Attachment — Submitted filename: Response to Reviewers (2) PLOS.docx [file pone.0337968.s006.docx]

**We thank Reviewers 1 and 2 for their substantive feedback and Reviewer 3 for their new feedback. We have addressed the comments and concerns from Reviewer 3, detailed below.**

**Reviewer #1:** No further comments from my side, thank you for the opportunity to review this paper. great job and congrats

**Reviewer #2:** The authors addressed my comments and improved the overall clarity of the paper. I especially appreciated the author's clarifications of their statistical approach choices in their response to the comments document.

**Reviewer #3:** This manuscript examines whether violent deaths change in the three months after federally declared disasters across five U.S. states, using NVDRS data and FEMA public assistance eligibility as the exposure proxy. The overall framing and use of injury date for event timing are appropriate for an acute-effects question. Several methodological clarifications would strengthen rigor and interpretability.

Abstract

1. Explicitly name the design as an ecological, quasi-experimental pre/post comparison with internal controls and state fixed effects. This prevents readers from over-inferring causal claims.
   - This is now included in the Methods section of the Abstract. This section now reads: “This study uses an ecological, quasi-experimental design to assess violent deaths in pre-disaster and disaster periods across five U.S. states (e.g., North Carolina, Oregon, Oklahoma, Wisconsin, and Colorado) with federally declared disasters resulting from natural hazards (e.g., flood, wildfire, tropical cyclone, storms). Deaths recorded in the National Violent Death Reporting System were used to describe violent deaths with injuries occurring three-months prior to and after disaster onset. Poisson regression with population offsets and fixed effects was used to calculate incidence rate ratios for disaster affected and unaffected counties within the same state, comparing violent death rates in disaster periods with non-disaster periods.”
2. Specify the geographic scope and hazard types in a single sentence to anchor external validity.
   - Please see above response to comment 1). The Methods section of the Abstract now reads: “ This study uses an ecological, quasi-experimental design to assess violent deaths in pre-disaster and disaster periods across five U.S. states (e.g., North Carolina, Oregon, Oklahoma, Wisconsin, and Colorado) with federally declared disasters resulting from natural hazards (e.g., flood, wildfire, tropical cyclone, storms).”
3. Report the main effect sizes with 95% confidence intervals for affected and unaffected areas. Briefly note any state-level heterogeneity so readers understand that pooled nulls can mask contrasts.
   - Additional clarification has been added in the Results: “Rate ratios were heterogenous but not significant across individual disasters, with only non-suicide and all-cause violent deaths increasing significantly following severe storms and flooding in Wisconsin for counties ineligible for public assistance.”

Introduction

1. Tighten the rationale for the three-month window by linking it to hypothesized acute stress pathways and distinguishing it from longer recovery windows that may show different effects.
   - This has been addressed in the Study description and population section of the Materials and methods, with an additional sentence reading: “The three-month disaster period was selected to measure acute disaster-related mental health impacts rather than more longitudinal impacts outside of the immediate response period.”
2. Justify the choice of FEMA public assistance eligibility as the exposure surrogate. Briefly discuss its relationship to hazard intensity and potential misclassification.
   a. We added a sentence describing the rationale for using FEMA public assistance eligibility as a proxy for disaster exposure, which now reads: “FEMA public assistance eligibility was used as a proxy measure for disaster exposure because federal assistance eligibility requires a community to have loss and damage that surpasses local and state-level capacity to address resulting acute needs, response, and recovery. It is important to note, however, that smaller scale disasters that are not granted federal assistance can still have tremendous effects on impacted communities, and, even within those eligible for public assistance, there is appreciable variation in need.”
3. Situate the work within prior disaster mortality literature that has focused mainly on suicide. Explain why including non-suicide violent deaths adds value and what you expect to see a priori.
   - We have added a sentence to the final paragraph of the Introduction, reading: “By assessing deaths resulting both from suicides and interpersonal violence, this study adds to the literature base for understanding sequelae of violence in disaster contexts.”
   - We have added an additional phrase in the final paragraph of the Introduction, which now reads: “Given these complexities, it is critically important to consider the mental health impacts of disasters, including not only violent behaviors but also violent deaths.”

Methods

1. Design and unit of analysis. State plainly that this is an ecological pre/post design with within-state internal controls. Clarify the analysis unit used in models (aggregated eligible vs ineligible counties within state, or county-level panels).
   - Additional clarification has been added in the Model specification for incidence rate ratios section of the Materials and methods, reading: “This study uses an ecological pre-post (i.e., pre-disaster and disaster period) design with within-state internal controls (i.e., affected an unaffected counties).”
2. Time windows and dating. Define how the three-month pre and post periods are constructed relative to the disaster start date. Confirm that assignment uses injury date rather than death date.
   - This is stated in the sentence including the phrase, “injury data (rather than death date),” but an additional sentence has been added to further clarify this rationale: “Using injury date ensures that the causative injury event preceding a violent death occurred in the temporal range of interest.”
   - Three-month pre and post periods are now shown in Table 1 to clarify how these periods were constructed. An additional sentence has been added to read, “The first date of the disaster period is the date of event onset.”
3. Exposure definition. Describe whether FEMA eligibility is county-wide or jurisdiction-specific within counties. Acknowledge cross-boundary injuries and consider a sensitivity analysis assigning exposure by injury county as well as residence.
   - This is discussed in the Limitations section: “We used eligibility for federal public assistance to determine if a county was disaster affected. However, the effect of a disaster on a community is considerably more nuanced than the presence or absence of a federal disaster declaration. For example, populations within a community could still experience highly disparate disaster impacts from one another, underscoring the importance of capturing disaster exposure on a more granular scale.”
4. Outcome mapping. List the mapping from NVDRS intents to your three outcomes: all violent deaths, suicide, and other violent deaths. Make it reproducible enough to be replicated.
   - The following text has been added in the Outcomes section: “ICD-10 codes corresponding to intentional self-harm or sequelae of intentional self-harm were used to determine suicide deaths. All other deaths were considered ‘non-suicide violent deaths.’ All violent deaths include any death recorded in the NVDRS database, including both suicide and other forms of violent deaths.”
5. Model specification. State the exact model formula, link, offset, and software. Clarify whether the offset reflects population or population-time for the three-month window. Report checks for overdispersion and whether you considered negative binomial models or robust standard errors. Specify how standard errors were clustered.
   - These specifications are found in the Analysis and Model specification for incidence rate ratios subsections of the Methods. An additional sentence has been added to the Model specification for incidence rate ratios section, which reads: “Overdispersion was assessed by comparing the mean and variance of the count data, and Poisson regression was an appropriate modelling selection.”
6. Covariates and seasonality. Explain why demographic variables are used descriptively rather than in models, given the ecological unit. Address seasonality either by month-matching pre/post windows, including calendar-month fixed effects, or using a county-month panel as a sensitivity analysis.
   - Given small sample sizes, it was not possible to stratify by race, sex, marital status, or education level. We have clarified by adding the sentence, " Because the unit of analysis for this study was the aggregate of affected and unaffected counties, sociodemographic characteristics are reported to contextualize the underlying population but are not included in regression models,” in the Measures section of Materials and Methods.
7. Sample selection. Provide a table listing events per state, the number of affected and unaffected counties, and the population denominators used in each period.
   - Please reference Table 1.
8. Missingness. Report missingness for all descriptive variables. Even if not modeled, patterns of missing data can inform interpretation of group differences.
   - There was no appreciable missingness for descriptive variables, with missing variables for education shown in Table 3.

Results

1. Present crude rates per 100,000 with 95% confidence intervals alongside IRRs for the main contrasts. This grounds effect sizes in absolute terms.
   - These crude rates are presented in supplemental files.
2. State clearly whether overall IRRs differ between pre and post periods for affected and unaffected groups, and do the same for suicides versus other violent deaths. Quote the IRRs and CIs to prevent over-interpretation.
   - IRRs are stated in the Results section paragraph following the in-text place holder for Table 3.
3. Summarize heterogeneity with care. Highlight where non-suicide violent deaths or suicides deviate from the pooled pattern, and pair these statements with uncertainty intervals.
   - All findings are null, although direction from the null differs. The only deviation from this pattern is described in the Results paragraph following the in-text marker for Table 3, reading: “Only Wisconsin saw a significant increase in non-suicide violent deaths for unaffected counties (IRR: 2.522; 95% CI: 1.600 – 3.979), which drove the observed increased rate of all violent deaths in the disaster period for unaffected counties in Wisconsin.”
4. Where cell suppression prevents detailed tabulation, consider a forest plot of state-specific IRRs with confidence intervals for each outcome group to communicate patterns without disclosure risk.
   - We appreciate the Reviewer’s suggestion and, after creating forest plots, have selected to continue with tables to most effectively show results. At the discretion of the editor, forest plots can be submitted as Supplemental files.

Discussion

1. Calibrate language to the study design. Replace causal terms with associational phrasing. Emphasize that the ecological, pre/post design cannot establish causality.
   - We have added explicit language around study design and limitations throughout the entirety of the manuscript to ensure that readers do not interpret any results to be causal in nature.
2. Expand limitations on exposure misclassification from using FEMA eligibility, cross-boundary injuries, seasonality, and the loss of information from aggregating counties into affected and unaffected groups. Discuss likely directions of bias.
   - Please find this concern addressed in the Limitations section, where we discuss the limitations and potential biases resulting from the selected timeframe, county-level aggregation, cross-border effects, and the use of FEMA public assistance eligibility for disaster exposure.
3. Interpret pooled nulls in light of heterogeneous state-specific results and the focus on acute, largely non-catastrophic events. Clarify that findings may not generalize to catastrophic disasters or to longer recovery periods.
   - We have added text noting that catastrophic (e.g., high direct mortality) events could result in different observed violent death rates, given that witnessing deaths is an indicator of negative mental health outcomes, particularly post-traumatic stress disorder.
4. Outline next steps: a county-month panel with county and month fixed effects, event-study graphs to check pre-trends, exposure sensitivity using injury county, and alternative count models if overdispersion is present.
   - We have specified several next steps, including studies accounting for the role of displacement and sheltering in the relationship between disasters, stress, and violent deaths and studies evaluating the utilization and effectiveness of mental health programs in disaster affected communities. We also outline the potential biases and limitations resulting from the use of aggregate units of analysis. Finally, we state that future studies should aim to assess the causative relationship of disasters on suicide and violent death mortality rates.
5. Overall, the study is well-motivated and pragmatically designed for an acute-effects question. Clearer design labeling, fuller model diagnostics, explicit handling of seasonality, and results visualizations that foreground uncertainty would materially strengthen the manuscript’s rigor and usefulness.
   - We thank the reviewer for their substantive comments, and we believe we have adequately addressed concerns, thereby strengthening the manuscript.
